# Squamous Cell Carcinoma Arising from an Oral Lichenoid Lesion: A Case Report

**DOI:** 10.5681/joddd.2012.007

**Published:** 2012-03-13

**Authors:** Ali Taghavi Zenouz, Masoumeh Mehdipour, Rana Attaran, Ayla Bahramian, Paria Emamverdi Zadeh

**Affiliations:** ^1^Associate Professor, Department of Oral Medicine, Faculty of Dentistry, Tabriz University of Medical Sciences, Tabriz, Iran; ^2^Assistant Professor, Department of Oral Medicine, Faculty of Dentistry, Tabriz University of Medical Sciences, Tabriz, Iran; ^3^Post-graduate Student, Department of Oral Medicine, Faculty of Dentistry, Tabriz University of Medical Sciences, Tabriz, Iran; ^4^Assistant Professor, Department of Oral Pathology, Faculty of Dentistry, Tabriz University of Medical Sciences, Tabriz, Iran

**Keywords:** Oral lichen plan, lichenoid reaction, squamous cell carcinoma

## Abstract

Lichenoid reactions represent a family of lesions with different etiologic factors and a common clinical and histologic ap-pearance. Lichen planus is included with lichenoid reactions and is a relatively common chronic mucocutaneous disorder. The most important complication of lichenoid reactions is the possibility of malignant transformation. That is why it has been considered a precancerous condition. Although the malignant transformation rate varies widely in the literature, from 0.4 to 6.5 percent, in most studies it does not exceed 1%. The aim of this paper is to report a rare case of squamous cell car-cinoma (SCC) arising within an oral lichenoid lesion in a 17-year-old woman, where SCC is very uncommon. The patient did not have any risk factors and was healthy. The lesion was located on the border of the tongue. In view of thecommon occurrence of OLP (oral lichen planus) and the unresolved issues regarding its premalignant potential, this case report illus-trates the need for histologic confirmation and a close follow-up of clinical lesions with lichenoid features.

## Introduction

Lichenoid reactions represent a family of lesions with different etiologic factors but common clinical and histologic appearance.Lichen planus as a subtype of lichenoid reactionsis a relatively common chronic mucocutaneous disorder.^1^Studies have indicated that oral lichen planus (OLP) occurs in 0.5-2.2% of the population, with a peak incidence in the third to sixth decades of life, with twice as many affected women as men.^2^ Several clinical subtypes have been recognized, including reticular type, papular type, bullous type, plaque type, and ulcerative type.^3^

Lesions are characteristically bilateral, commonly involving the buccal mucosa, tongue, gingiva, palate, floor of the mouth or lips.^4^ Asymptomatic, bilaterally symmetrical reticular OLP affecting the buccal mucosa is the most common oral presentation.^3 , 5 , 6^

In addition, in a patient with oral lesions, extraoral areas, such as the anogenital, conjunctival, esophageal or laryngeal mucosa might be involved.^4^

The most important complication of lichen planus is the possible malignant transformation; therefore, it has been considered a precancerous condition.^7^ A 1% incidence rate of squamous cell carcinoma has been reported among patients with this condition in both retrospective and prospective cohort studies. ^3^ However, the true risk remains controversial, given the heterogeneous diagnostic criteria for lichen planus across studies (and the difficulty in discriminating it from other premalignant conditions), the variation in the duration of follow-up periods, and the potential confounding by associated risk factors (e.g. alcohol consumption and smoking).^3 , 8^Case reports have also described squamous cell carcinomas arising from chronic oral, anogenital, esophageal, or hypertrophic cutaneous lichen planus lesions.^9^

While some experts believe in an innate malignant transformation capacity for OLP, others claim that only lichenoid lesions with dysplasia—referred to as lichenoid dysplasia or LD—are potentially cancerous.^2 , 3^

Additional prospective clinical studies with strict clinical and histopathological criteria for the definition of oral lichen planus are necessary to answer this question.^10^

The aim of this paper is to report a case of squamous cell carcinoma (SCC) arising within an oral lichenoid lesion in a very young patient, where SCC is very uncommon.

## Case report

A 17-year-old white female patient, housewife, living in Shabestar (Northwestern Iran), referred to the Department of Oral Medicine, Faculty of Dentistry, Tabriz University of Medical Sciences, in November 2010 with the chief complaint of a tongue lesion for the previous 6 months. She had been visited by a general practitioner, who had prescribed iron and folic acid supplements, which had resulted in no significant changes in disease process.

**Figure 1:**
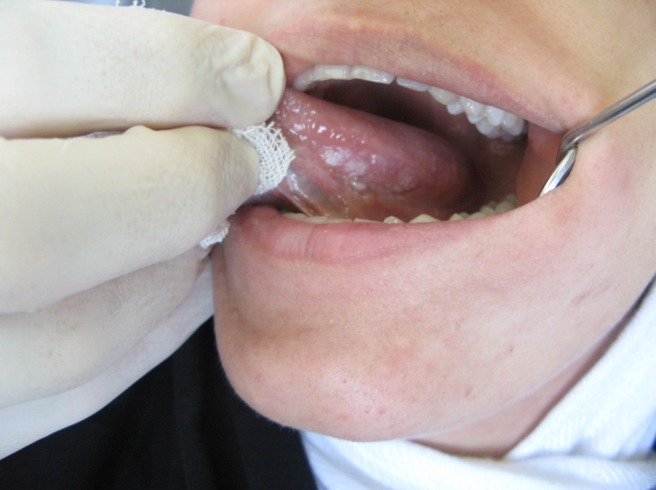
White keratotic plaque on the left lateral border and ventral aspect of the tongue.

Upon physical examination, a keratotic plaque was observed on the left lateral border and ventral aspect of the tongue with the largest diameter of 1.8 cm and irregular borders. There were two discrete white papules on the dorsal aspect of the lesion located posterior to the plaque. An erythematous and atrophic area was located on the ventral aspect of the tongue and there was a small ulcer distal to the main lesion (white plaque) (Figure 1). Borders of the ulcer were indurated and non-tender. No abnormality was detected in cervical lymph nodes on clinical manipulation. In medical history a microcytic hypochromic anemia was noticed. The patient denied history of smoking, alcohol consumption or any other harmful habits.

**Figure 2:**
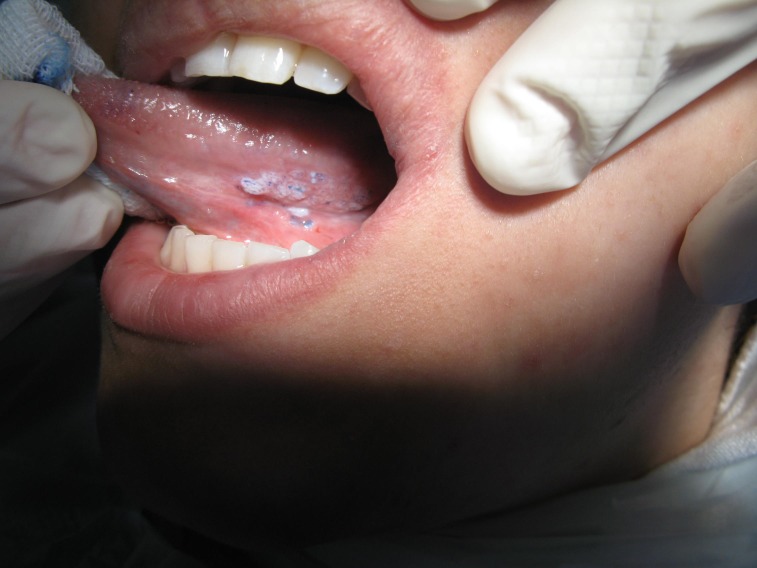
Discrete positive staining with Toluidine Blue.

Formulated diagnostic hypotheses based on clinical findings were those of lichenoid reaction and SCC arising from lichenoid reaction. Toluidine blue staining was applied and discrete positive staining was observed (Figure 2).

An incisional biopsy was carried out; in histopathologic examination, pleomorphism, nuclear hyperchromatism, individual cell keratinization, invasion to stroma, hydropic degeneration of basal cells layer and heavy bandlike infiltration of chronic inflammatory cells were observed (Figure 3). Histopathology confirmed squamous cell carcinoma.


Figure 3. Photomicrograph of lichenoid reaction showing degeneration of the basal epithelial layer and an intense lymphocytic infiltrate subjacent to the epithelium (left); photograph of SCC showing dysplastic epithelial cells with hyperchromatic nuclei and individual cell keratinization invading into the stroma (right).

**Figure:**
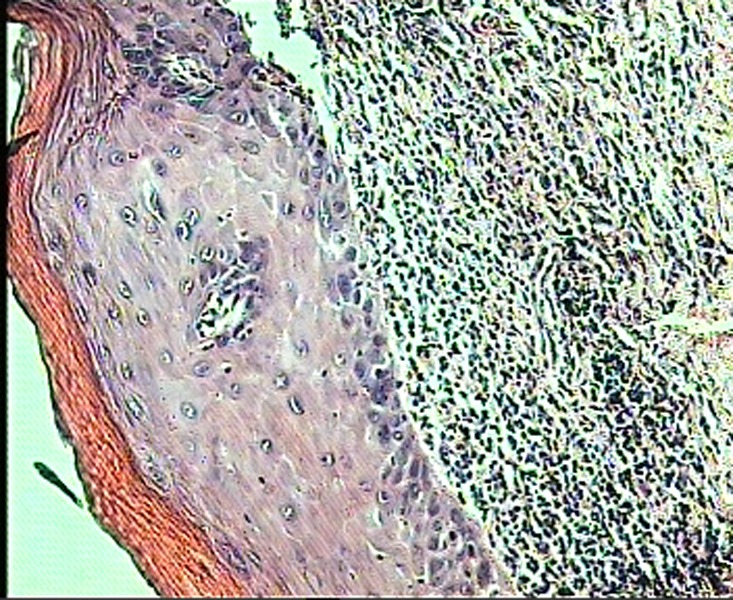

**Figure:**
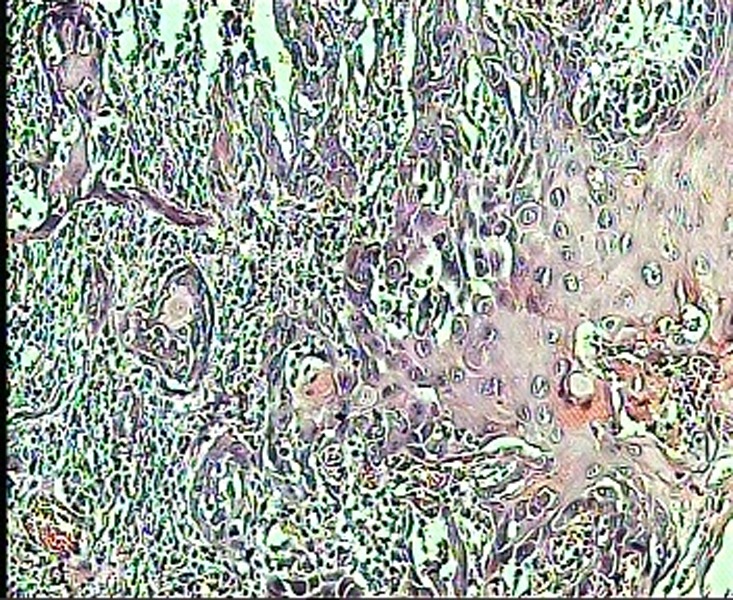

After incisional biopsy, liver and spleen sonography and chest x-ray were requested and no abnormality was reported. In spiral CT scan of the tongue, floor of the mouth and neck, two lymph nodes with short axis of 5 mm were seen in submental area without cervical lymphadenopathy. Deep muscles of the tongue were normal. As treatment plan, hemiglosectomy with the neck dissection in I, II, III, and IV surfaces was carried out. Histopathologic result was: eight normal reactive lymph nodes in the cervical mass; residual tongue mass; lymph nodes free of tumor; and mild to moderate dysplasia in the floor of the tongue mass.

In this case, SCC was classified as T1N0M0 (stage I), based on the oral cancer TNM classification criteria of the UICC/AJC (American Joint Committee for Cancer Staging).^11^

The patient is currently under periodic follow-up. No recurrence has been detected until April 2012.

## Discussion

One of the hallmarks of OLP is the intense inflammation seen in the underlying connective tissue. There is association between chronic inflammation and an increased risk of the malignant development.^12^

The World Health Organization (WHO) classifies OLP as a potentially malignant disorder with an increased risk of developing into squamous cell carcinoma of the head and neck region (SCCHN). The malignant properties of OLP lesions are somewhat controversial and there are different opinions about the premalignant character. Some studies have shown the increased risk of developing squamous cell carcinoma in OLP lesions compared to the general population. The reported percentage of tumor development is 0.07-5.3%. There are also no reports about malignant transformation of OLP lesions.^13^

The lack of accepted specific diagnostic criteria of OLP might contribute to variations in the frequency of reported malignant transformation of OLP. The location most often affected by tumors in OLP patients seems to be the tongue and the buccal mucosa.^12 , 14^ Authors havereported a higher risk for malignant transformation in women with OLP than men with OLP.^12 , 14^

Researchers have not found tobacco and alcohol exposure to be greater in patients with OLP compared with the general population.^2^A number of investigators have reported a high degree of malignant transformation in homogenous, plaque-like and reticular OLP lesions, advocating their close follow-up, particularly when they involve cancer-prone sites in patients with high-risk behavior, i.e. smoking and drinking. ^4 , 5 , 13^ Our patient did not report any history of exposure to tobacco or alcohol.

There are several oral lesions that are clinically very similar to or even indistinguishable from OLP but they have a distinct etiology. Therefore, the diagnosis of OLP should be based on both clinical and histological criteria.^15^

If multiple biopsies are not feasible, it is best to perform a biopsy of the lesion involving the cancer-prone location.^6^ Moreover, biopsy site selection is critical because the histologic features might vary within the lesions, and cytologically significant areas might be missed or overlooked. Ulcerated, indurated or exophytic areas are most likely to demonstrate dysplastic or malignant features; therefore, clinicians should choose them for sampling.^2^

The use of test methods such as vital staining with toluidine blue and brush biopsy (cytology) may enhance the possibility for identification of signs of dysplasia or carcinoma in existing lesions.^16^ It is postulated that an increased amount of DNA and RNA in neoplastic cells and the wider intercellular canals compared to normal epithelial cells are responsible for staining malignant cells. While screening for recurrence in patients who had previously been treated for upper aero-digestive tract malignancies, it has been found that the use of toluidine blue in an unaided clinical examination increased the sensitivity of detecting a malignant neoplasm from 26.6% to 96.7%. Its ability to detect malignant oral cavity lesions has been well documented in clinical settings.^1^ It remains to be shown if these methods can be used as prognostic tools to identify OLP patients susceptible for future squamous cell carcinoma (SCC) development.^11^However, toluidine blue provides guidance for the selection of biopsy sites and improves the decision-making process to biopsy.^1^

In our patient, no causative agent which can produce lichenoid reaction was found. According to these data, histopathologic studies and clinical findings, the oral lichen plan (OLP) with neoplastic transformation (SCC) was the definitive diagnosis.

SCC is not frequent in young patients. Only 1-6% of SCC cases occur in patients under the age of 40; the occurrence in children and adolescents is extremely rare. Characterization of young patients with head and neck SCC is arbitrary. Most authors consider the young patients with SCC as those less than 40 years of age, even though others use as reference ages under 20 or 30 years. Age average in cases registered in literature as young bearers of SCC ranges from 30.8 to 34.2, with the largest part of the patients belonging to male gender.^18^

However, the association between some types of anemia such as sideropenic anemia (Plummer-Vinson Disease),^1^ Fanconi’s anemia,^19^ and SCC^1^has been reported. Our patient suffered a mild iron deficiency, which might not be an etiologic factor for SCC.

## Conclusion

In view of both the common occurrence of OLP and unresolved issues regarding its premalignant potential, this case report illustrates the need for histologic confirmation and close follow-up of patients with clinical lesions that have lichenoid features.^1^

It is mandatory to schedule meticulous long-term follow-ups, even in patients with asymptomatic or barely symptomatic oral lesions of lichen planus.^20^
